# Skeletal facial balance and harmony in the cleft patient: Principles and techniques in orthognathic surgery

**DOI:** 10.4103/0970-0358.57196

**Published:** 2009-10

**Authors:** Kenneth E. Salyer, Haisong Xu, Jason E. Portnof, Akira Yamada, David K. Chong, Edward R. Genecov

**Affiliations:** 1 World Craniofacial Foundation, Dallas, Texas, U.S.A; 2 Department of Biomedical Sciences and Department of Orthodontics, Baylor College of Dentistry, Texas A&M Health Science Center, Texas, USA; 3 Department of Plastic Surgery, Renji Hospital, Shanghai Jiao Tong University School of Medicine, Shanghai, China; 4 Department of Plastic and Maxillofacial Surgery, Royal Children's Hospital, Melbourne, Australia, Texas, USA; 5 Department of Orthodontics, Baylor College of Dentistry, Texas A&M Health Science Center, Dallas, Texas, USA

**Keywords:** Cleft lip and palate, Orthognathic surgery, Skeletal facial balance

## Abstract

The management of the palatal cleft, dental arch, and subsequent maxillary form is a challenge for the craniomaxillofacial surgeon. The purpose of this paper is to present the experience of a senior surgeon (KES) who has treated over 2000 patients with cleft lip and palate. This paper focuses on the experience of a recent series of 103 consecutive orthognathic cases treated by one surgeon with a surgical-orthodontic, speech-oriented approach. It will concentrate on not only correcting the occlusion, as others have described, but also on how a surgeon who was trying to achieve optimal aesthetic balance, harmony, and beauty, approached this problem.

## INTRODUCTION

Facial balance and harmony are very important in society's acceptance of an individual. The cleft deformity frequently results in abnormalities that must be treated from the time of birth until facial growth is completed. Skeletal surgery is an important and integral part of cleft habilitation. The final surgery cannot be performed until after growth is completed. In the senior author's (KES) experience using a surgical-orthodontic protocol, 30–40% of the patients required orthognathic surgery to achieve optimal facial balance, occlusion, and normal speech. This paper focuses on the experience of a recent series of 103 consecutive orthognathic cases treated by one surgeon with a surgical-orthodontic, speech-oriented approach. The speech results have been reported recently in the literature for a portion of this series of patients.[1]

This paper will concentrate on not only correcting the occlusion, as others have described, but also on how one surgeon who was trying to achieve optimal aesthetic balance, harmony, and beauty, approached this problem. The primary planning was an ongoing process undertaken by the orthodontist and surgeon, who made the definitive planning just before the final operative correction.

## METHODS

### Surgical orthodontic planning and principles

Passive orthodontic treatment was used in infancy in this series of patients. The treatment starts within a few days after birth with an expansible acrylic appliance. In infants with maxillary collapse, expansion is used to reposition the maxillary segments and create a more normal alveolar arch. Following lip/nose repair, it is important to maintain the alignment of the maxillary segments. For this purpose, the appliance is worn until palatoplasty is performed at the age of eight months. Cancellous bone grafting was performed at the time of tooth eruption in the defect of the alveolus. Prior to that time, orthodontic expansion was performed at about 5½ years of age, and maintained until bone grafting was performed. Subsequent ongoing surgical orthodontic treatment was continual in these cases as it was deemed necessary according to our protocols [Tables 1 and 2]. Orthodontic treatment dictated an attempt to maintain as good an occlusion as possible during growth. This emphasis resulted in less definitive, late occlusal discrepancies in the mandible and maxilla.

**Table 1:** Orthodontic Treatment Protocol

*Patient age/stage*	*Treatment*
Two weeks	Passive infant appliance
5.5–8.5 years	Palate expansion
7–9 years	Preparation for bone graft
5–10 years	Face mask < 4 mm, Class III
Mixed dentition	Routine orthodontics
14–16 years 30% unilateral,	Final treatment,
40% bilateral	Perisurgical orthodontics

**Table 2:** Surgical Treatment protocol

*Patient age/stage*	*Treatment*
Three months	Primary cleft lip and nose
Eight months	Two-flap palatoplasty
Five years 35%	Secondary minor lip and nose surgery
7–9 years 100%	Cancellous iliac bone graft to alveolar cleft
Seven years-Full Growth	Distraction osteogensis in selected severe cases > 12 mm, Class III
Full growth – 30% unilateral, 40% bilateral	Orthognathic surgery
8–18 years	Rhinoplasty-other soft tissue

In clinical situations where there was a minimal (up to 4 mm) class III discrepancy, the Delair facemask was used to improve the occlusion. At the time of this treatment that was performed during the mixed dentition stage, it was not possible to predict which cases would ultimately need orthognathic correction. An attempt was made to correct the maxillary deficiency that was present at the time of cancellous bone grafting. The cleft maxilla grows abnormally due to the cleft dysmorphogenesis and the inherited growth pattern of each particular patient, in addition to the influence of surgical scarring. These factors compound and contribute to the maxillary deficiency that is inherent in the cleft patient. The search goes on for improved treatment protocols that will produce an optimal outcome of speech, occlusion, and facial balance. We prefer an attractive face, not just deformity correction.

Prior to the completion of growth, these patients were treated with ongoing orthodontics with alignment and leveling of the teeth. Patients who had occlusal discrepancies, were treated in preparation for orthognathic surgery. Definitive surgical planning was done when growth was complete, as determined by wrist X-ray films. A projecting facial skeleton with a full upper lip, showing upper six teeth when smiling, was a treatment goal.

The majority of the cleft patients that we treated were either our own patients from the beginning, or secondary cases that came to us having initial surgeries performed by other surgeons or teams. All patients presented with similar deformities. One of the major problems presenting in a unilateral cleft patient was the small and hypoplastic lesser segment which contributed to occlusal discrepancies with a cant that can only be treated adequately with jaw surgery. The vertical skeletal deficiency can not be corrected with orthodontic treatment alone.

The unilateral, vertically deficient lesser segment was one of the most difficult deformities to correct. Achieving an aesthetic balance and harmony of the face was arduous in these situations. When we first started doing this work, internal rigid fixation did not exist. Stabilization of a one-piece maxillary advancement required overcorrection by 20%. In later treatment, with the availability of internal rigid fixation, we did not have to perform overcorrection. In this environment, we started doing multiple piece correction with improved stability and decreased relapse. Noncleft patients undergoing similar procedures by the same surgeon, experienced a much lower relapse rate.

Presurgical planning involved both the orthodontist and surgeon examining the patient together in consultation. It was in this evaluation session that the exact postoperative position of the maxilla was determined. This maxillary position would be decided by optimal facial balance and harmony of each individual patient. Overcorrection of the maxilla was planned according to the upper lip deficiency and the amount of tooth show. The future vertical height of the maxilla was determined by the amount of incisal display with the patient smiling. The goal was 2–3 mm of tooth display on smile with adequate overjet. A healthy dentition and a beautiful smile are important in overall beauty and projection of the face.

During the planning of facial reconstruction, the main emphasis was on facial aesthetics and in this respect, it was not occlusion-driven surgery, but rather, facial-guided surgery that we embraced. The goal for surgical planning was to achieve an optimal three-dimensional positioning of the maxilla, which was the keystone in the planning of the facial projection and aesthetic balance.

Cephalometric tracings, dental models, occlusal wafers, and other planning methods were utilized. Adjunctive planning methods included computer software packages that enabled computer planning, supplementing the eye of the surgeon and orthodontist in determining optimal facial balance and harmony. The senior surgeon (KES) prefers the creation of a convex facial skeleton. Facial convexity and projection are very important in overcoming the stigmata of the cleft deformity, and were key goals of the senior surgeon in achieving optimal facial beauty. Maxillary projection, malar angularity with prominence, and proper show and projection of the upper teeth are important. The occlusion is then corrected by mandibular repositioning and possible genioplasty to create vertical and sagittal facial balance.

Once the position of the maxilla was determined, the mandible was placed according to the maxilla's position. In this series of patients, at least half received a bilateral sagittal split of the ascending ramus of the mandible. A sliding genioplasty is an important consideration to achieve improvement of the facial balance and to optimize the projection and balance of the face. Very frequently, the senior surgeon would make the final decision on performing the genioplasty at the time of the surgery, after placing the maxilla and mandible in their proper occlusal positions using prefabricated occlusal splints. The genioplasty provided the opportunity to not only achieve projection of the mandible with a balancing of the vertical height of the chin, but could also allow adjustment of the lower third of the face, when necessary.

After achieving a proper maxillary position and moving the mandible so that the occlusion is corrected with the now-projecting facial skeleton, attention is then directed to the other major component in achieving facial balance and harmony: the projection of the malar cheekbones. In this series of 103 patients, the senior surgeon chose to achieve projection of the malar bones using perforated, demineralized bone. It was found to be safe and produced a consistent and aesthetic result, with minimal resorption.

Demineralized bone was easy to use, and the senior surgeon had an extensive experience with this material (1990 to present).[2–4] During this period, over 1000 implants of various forms were used from one bone bank (Pacific Coast Tissue Bank, Los Angeles, California). The perforated, demineralized bone implants were placed via the intraoral approach at the time of the surgical correction in over half of the patients in this series. These implants provided the added fullness to the malar region needed to augument, provide projection and angularity to the face, and improve aesthetic harmony.

In this series of patients receiving perforated, demineralized bone onlay grafts, none experienced any significant complications. Perforated, demineralized bone is an osteoinductive, biocompatible material that was well accepted by the patient and underwent remodeling with minimal resorption of the implant. In fact, the results were so good that no other technique in this series of 103 patients was used to achieve projection of the malar region in front of the maxilla. The other techniques that have been discussed in this paper were used for patients with other diagnostic problems. The lamellar split technique[5,6] is not used in combination with a Lefort I operation, but is included in this paper as an appropriate reference for augmentation surgery of the malar region.

The correction of the facial skeleton, including the occlusion, is based on the optimal position of the maxilla. It is this position of the maxilla that then sets up the foundation for the remaining facial skeleton. The cleft deformity results in a deficient upper lip and vermillion necessitating the facial skeleton and the teeth to be overcorrected in these cases. Creation of aesthetically pleasing facial curves with balance of the cheeks, the upper jaw with the teeth, the lower jaw with the teeth, and a solid, stable occlusion are important for facial balance.

The other key component in achieving an attractive face is the correction of the nose. During the entire career of the senior surgeon, emphasis was always placed on the primary and secondary correction of the cleft nasal deformity.[7,8] The secondary correction of the nose, septum, turbinates, and other paranasal skeletal deformity contributed to achieving an attractive face with normal function. All of these elements are important in achieving excellence in the surgical reconstruction of the cleft patient. Proper attention to the nasal malformation is how the senior surgeon has provided extensive, safe, and consistent aesthetic results in cases of cleft deformity.

### Maxillary lefort I surgical technique

Bovie monopolar electrocautery with a Colorado micropoint needle is preferred for mucosal incisions. Incisions are made in the maxillary gingivobuccal sulcus 3–4 mm above the upper gingiva. A cuff of mucosa is preserved to facilitate closure. If an alveolar fistula is present, incisions are made along the cleft margin to allow two-layer closure of the fistula. In a bilateral cleft case, care is taken to preserve a labial cuff of mucosa on the premaxilla (especially in the presence of preexisting fistulae).

Blunt dissection with a periosteal elevator exposes the maxillary surgical site. Care is taken not to expose the buccal fat pad and to preserve the bilateral infraorbital nerves. The nasal mucosa is dissected from the bony floor of the nose and this nasal mucosal lining is protected with a ribbon malleable retractor during the maxillary bony cuts.

Lefort I level maxillary osteotomies are made 4–5 mm superior to the root of the first bicuspid tooth. Anterior maxillary osteotomies are made with a reciprocating saw, which is also used for bone cuts through the maxillary tuberosity and the posterior wall of the maxillary sinus. If the posterior wall is too deep for the length of the saw, the osteotomy can be completed with a thin Dautrey osteotome. A bifid nasal osteotome is used to separate the vomer from the nasal septum and lining. A thin, straight osteotome is used to osteotomize the lateral nasal walls.

A stair-step modification of the Lefort I osteotomy can be used in cases of planned vertical maxillary shortening. The stair-step procedure allows adequate vertical reduction and locks the bony segments into position, ensuring secure bone-to-bone contact after advancement. Angling the osteotomy from posteriorly higher to anteriorly lower allows elongation of the maxilla modifying the Lefort I. Such a modification allows maxillary lengthening as the maxilla is brought forward.

Maxillary downfracture is created with either finger pressure or Rowe disimpaction forceps. After completion of the downfracture, the posterior wall osteotomy is inspected. If the osteotomy is incomplete, a Dautrey osteotome can be used in a controlled fashion to complete the osteotomy, without inadvertent fracture of the pterygoid plate.

#### Pterygomaxillary dysjunction

**Figure 1:**
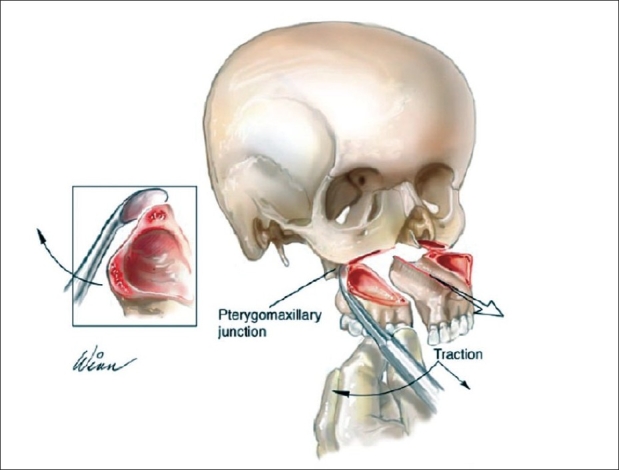
Technique for pterygomaxillary dysjunction

Pterygomaxillary disjunction and advancement are facilitated with a curved periosteal elevator, which is wedged into the pterygomaxillary suture and advanced. This technique creates less damage than an osteotome and can be utilized in almost every case [See Figure 1]. Surgical manipulation in this region is performed with extreme care to protect the branches of the internal maxillary artery.

The technique has been used for pterygomaxillary disjunction by the senior surgeon (KES) and has evolved as KES performed his work over a period of 40 years. Early experience with the pterygomaxillary disjunction involved a large osteotome (as used by Tessier) but was found not to be necessary to perform this procedure. Over time, in the 70s, the senior surgeon (KES) discovered that this procedure could be performed without an osteotome, thus improving the results and reducing morbidity by uncontrolled minimizing blood loss and avoiding pterygoid plate fracture, as seen with an osteotome.

The downfracture is the key maneuver before performing pterygomaxillary disjunction. It is necessary that the downfracture be performed cleanly and completely before attempting the disjunction with a curved periosteal elevator. Once the surgeon becomes adept at this technique, (s)he never will find it necessary to ever use an osteotome again. Blindness has been reported in the literature when disjunction was performed with an osteotome during a Lefort I osteotomy.[9] It is believed that this complication can be prevented by using a periosteal elevator instead of an osteotome. It is highly recommended that the surgeon learn this technique in order to improve operation safety and decrease blood loss.

**Figure 2:**
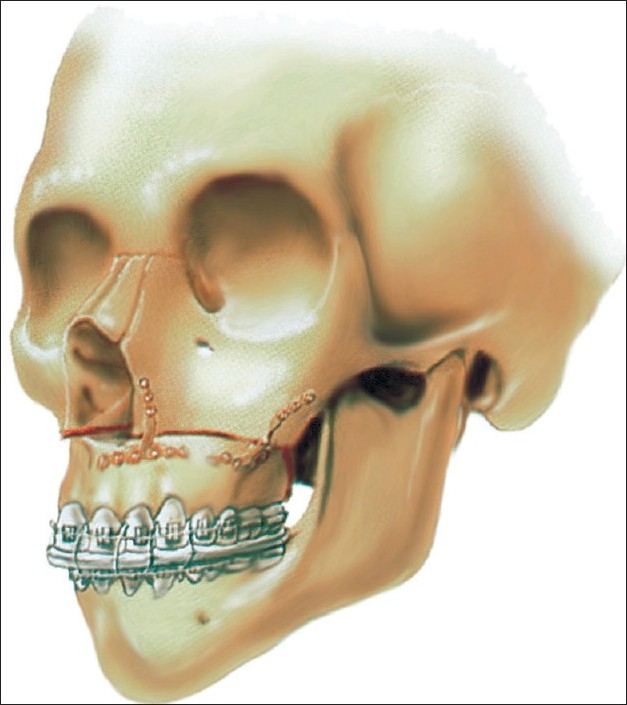
Internal semi-rigid fixation using titanium “T” plates

The maxilla is mobilized into the preplanned position by using wire fixation and adjusting the vertical height before being placed into temporary intermaxillary fixation (IMF) using a prefabricated occlusal splint. If bimaxillary surgery is planned, the splint will have a removable lower piece to differentiate the intermediate *vs* the final position of the mandible. Bony impingements to the planned maxillary movements are removed carefully with a burr or bone rongeurs. Plating of nasomaxillary and zygomaticomaxillary buttresses is then performed with titanium “T” plates designed by the senior author (KES) [See Figure 2]; two plates are to be used on each side.

If the maxilla requires differential movement or widening, controlled osteotomies are created following down-fracture with a reciprocating saw. A fine burr is used between the diverging roots of teeth. The orthodontist will facilitate the osteotomy by angling the dental roots distally or mesially.

Cancellous bone is grafted if required, and the mucosa and gingiva are sutured closed. Care is taken to keep the nasal and oral layers intact so as not to jeopardize the bone graft. If necessary, the dental splint is removed temporarily to facilitate closure.

The alar base cinch is utilized to prevent nasal base widening after maxillary surgery. Patients undergoing advancement and/or superior repositioning of the maxilla are most prone to visual changes in the caudal aspect of the nose.[10]

The technique for alar base cinch begins with the identification of the ala base fibroalveolar connective tissue through the circumvestibular incision. A 3-0 or 4-0 absorbable suture is passed through the alar base and gently pulled medially. The suture is passed through the perinasal musculature laterally-to-medially. Positioning an index finger directly on the alar base will improve the sense of correct suture placement.[11] The ala is inspected extraorally to verify that the base has been accurately identified. An identical procedure is used to pass the suture through the opposite ala; thus, the suture is passed in a figure of eight pattern. Symmetric movement of both alae is verified and distortion from the nasotracheal tube is noted and taken into account during the final suture tightening; the suture is then tied.

The alar base width is measured with calipers and the measurement recorded prior to surgery. The alar base width is again measured postoperatively and compared with the preoperative value to ensure that proper surgical control of the alar base width has been maintained.

V-Y lip closure is used to prevent flattening and loss of vermillion of the upper lip. To perform the V-Y lip closure, a skin hook is inserted into the vestibular incision and used to retract the mucosal margin at the midline. The “Y arm” is created and closed with a continuous absorbable suture. The midline is re-approximated with interrupted absorbable sutures. The remainder of the incision is then closed with a continuous absorbable suture bilaterally. The continuous suture should be started posteriorly, and advanced toward the midline, pulling the superior tissue slightly forward. The VY lip closure of the vestibular incision is utilized to preserve lip fullness.

Patients are routinely placed into heavy intermaxillary elastics at the completion of the procedure and then transferred into the surgical intensive care unit for overnight monitoring. Nasal trumpet, nasogastric tube, and urinary catheter are utilized for optimal patient monitoring. These monitoring devices are removed before transferring the patients to the ward floor.

Often, patients will require additional, adjunctive procedures to address their skeletal facial deformity. Genioplasty and malar augmentation are utilized to create a convex face with aesthetic curvatures. It is the goal to construct projecting and symmetric cheek-bones, nose, and chin. Dermal fat grafts can be used to plump out the upper lip. Many adjunctive procedures are used to achieve an attractive face.

### Mandibular ascending ramus split osteotomy

**Figure 3:**
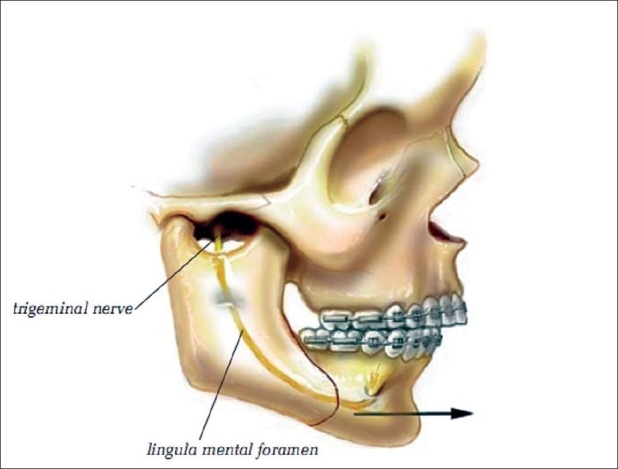
Mandibular ascending ramus split osteotomy. Note the important anatomical landmarks

The most important osteotomy of the mandible is the sagittal-split procedure originally developed by Trauner and Obwegeser.[12,13] Many modifications of this procedure have been described. The following technique is the one that the senior author (KES) has developed by using only a reciprocating saw to perform the osteotomy. We have found this procedure to be an excellent approach to a sagittal-split osteotomy. The disadvantage of all modifications is potential damage to the interior alveolar nerve which branches off the mandibular branch of the fifth cranial nerve. The mandibular nerve (V3), a branch of the trigeminal nerve, exits the brain via the foramen ovale at the base of the skull and then enters the mandible via the mandibular foramen at the lingula. It runs through the body of the mandible, exiting at the mental foramen as the mental nerve [Figure 3]. Panoramic and cephalometric radiographs, using the lingula and mental foramen as reference points, allow identification and visualization of the course of the nerve in the mandible. This information is essential to the surgeon while performing a sagittal-split osteotomy, a sliding genioplasty, and other mandibular osteotomies.

A bite block is inserted on the side opposite to where the surgery is performed. The surgeon stands on the right side of the patient and performs both the left-sided and right-sided osteotomies from that position. Identification of the anterior border of the ascending ramus is made intraorally by palpation with the surgeon's index finger. The initial incision in the oral mucosa is made lateral to the border by using a Colorado needle. A Bifurcated periosteal elevator is used along the anterior border of the mandible, stripping the periosteum of its muscle attachments up to the coronoid process, which is not shown in the drawing. A right-angled dental instrument facilitates identification of the mandibular notch, an important anatomical landmark. A bifurcated retractor is inserted high on the coronoid process and held by an assistant. By using a periosteal elevator, the lateral and medial subperiosteal planes are identified over the ramus and along the body of the mandible down to the angle. The J-shaped periosteal elevator is used, if necessary, at the lower inferior border of the mandible. The lingula and the inferior alveolar nerve are identified on the panoramic radiograph. Medially, the periosteum is freed above the lingula and below the sigmoid notch. Identifying the inferior alveolar nerve in isolation is not necessary. A lighted ramus retractor is inserted medially above the level of the lingula, resting on the posterior border of the ascending ramus.

**Figure 4:**
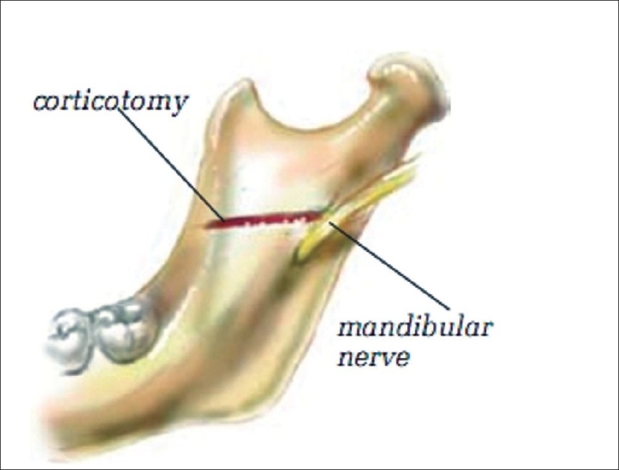
The corticotomy is carried through the inner cortical table of the ascending ramus to its anterior border above the nerve

The medial ramus corticotomy is performed with the retractor rotated and hooked behind the posterior border of the ramus. With a reciprocal saw, the corticotomy is carried through the posterior border of the ascending ramus of the mandible and along the inner cortical surface below the sigmoid notch and above the lingula and nerve. The corticotomy is carried through the inner cortical table of the ascending ramus to its anterior border above the nerve [Figure 4]. The medial ramus retractor may remain in place or be removed when retracting the mucosa anteriorly. With a reciprocal saw, the osteotomy is carried from the previous corticotomy, splitting the anterior border of the ascending ramus by placing the blade of the saw along the inner surface of the outer table. The remaining portion of the osteotomy is performed with retraction of the mucosa along the anterior border of the mandible. The osteotomy is continued through the ascending ramus and onto the body of the mandible. The saw blade cuts through the inner surface of the outer table. This osteotomy can also be performed by separating only the ascending ramus without bringing the cut anteriorly through the angle or the body of the mandible and has become the senior surgeon's preferred technique. The osteotomy is completed by sawing through the inferior border of the mandible. The saw is then brought through the outer table of the body of the mandible at a level at which the mandible can be advanced, maintaining contact between the bony segments. In cases in which the osteotomy is performed with a saw, the proximal and distal segments are easily separated by inserting a thin osteotome and rotating it to separate the two segments. When the two segments split with resistance, the osteotomy alone can be continued or a bone spreader is used, or both techniques can be utilized. The lighted suction is used to visualize between the outer and inner tables as they are separated to make sure the neurovascular bundle is in the medial part of distal segment. Further dissection of the periosteum and attached muscles is necessary in some cases to obtain proper separation of the bony segments.

**Figure 5:**
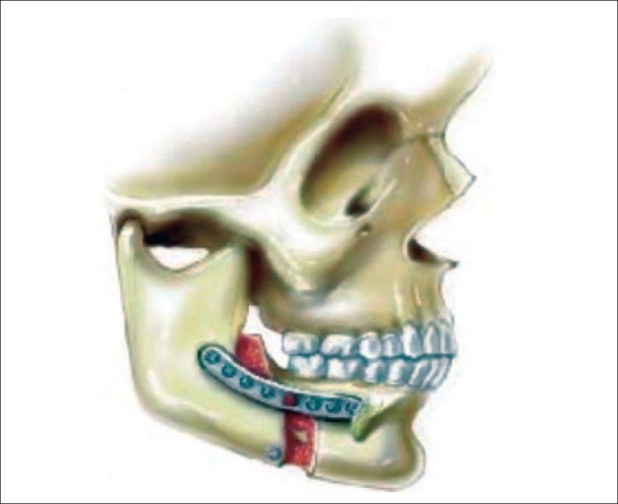
Rigid fixation is completed bilaterally

The fixation is begun by placing a single twisted wire between the proximal and distal segments in order to bring them together into their new position. It is important that the condyles maintain their positions in the temporal fossae. A prefabricated wafer is fixed to the teeth with fine orthodontic wires, and the upper and lower jaws are placed in temporary intermaxillary fixation. To fix the bony segment, titanium or cobalt-chromium (Vitallium) plates are used with a right-angled drill and screwdriver for screw insertion. The plate is bent to the contour of the mandible, following the shape of the template. Two holes are placed in the proximal segments, and two are placed in the distal segments. With a right-angled screwdriver, screws are placed through the outer and inner cortex of the bony segments [Figure 5]. The plate is fixed along the upper border of the proximal and distal bony segments. After the screws are inserted, the intermaxillary fixation is removed so that the mouth can be opened to check for any blockage of the movements. Elastics are used for postoperative intermaxillary fixation for six weeks and are removed when eating and exercising the temporomandibular joints postoperatively.

### Genioplasty–surgical technique

The most important single aesthetic unit of the lower third of the face is the chin. The chin can be moved independently from the remainder of the mandible in three planes of space with a sliding genioplasty. The chin can be surgically altered in contour and shape, and in position in both the horizontal and vertical dimensions. As the chin plays an important role in facial balance, genioplasty must be part of the armamentarium of the facial surgeon.

The first step in the genioplasty procedure is infiltration of a local anesthetic containing a vasoconstrictor. In order to prevent distortion of the shape of the chin, not more than 2 mL of solution should be infiltrated locally into the tissues.

The mandibular labial mucosal incision is made from a position that is distal of the canine to a similar point on the contralateral side. The incision is carried to the bone and the tissue is undermined subperiosteally. Special care is taken and a tunneling technique is utilized to preserve the bilateral mental nerves. A pedicle of tissue remaining around each mental nerve will protect against inadvertent avulsion. The lateral osteotomy is performed below the pedicle in a tunnel. The genial muscles remain attached to the chin. Degloving the chin is avoided, as it may result in redundancy or lack of tone of the lower lip.

The osteotomy for the sliding genioplasty is designed parallel to the occlusal plane in the anterior part of the inferior border of the mandible. If possible, the procedure should be carried out 4–5 mm below the mental foramen. The apices of the mandibular teeth and the bilateral mental nerves must be avoided during the bony incision. The osteotomy is carried as far posteriorly and bilaterally as possible to eliminate a step-off or indentation when sliding the genial segment forward and prevent a “button chin” which may not be aesthically natural-looking.

Prior to the anterior mandibular horizontal osteotomy, it is advisable to mark the dental midline and reference lines on the mandibular bone inferiorly and superiorly to the intended osteotomy. A cross-cut fissure bur may be used to score these reference lines.

The osteotomy is performed with a reciprocating saw while the mental nerves are protected by retractors.

After the chin is slid forward, it is fixed with three wires at three separate locations. These wires are placed through the outer table of the proximal segment to the inner table of the distal segment.

Either single-tiered or multiple-tiered sliding genioplasty can be accomplished. A two-tiered advancement is designed when 10–14 mm advancement is indicated. It is essential that the two osteotomies be parallel to each other and to the occlusal plane. Rigid internal fixation is required with a two-tiered advancement but rarely with a single-tiered.

Interpositional grafts of autogenous bone, hydroxyapatite, or demineralized bone can be employed when vertical augmentation of microgenia is indicated.[2–4,14] The use of rigid internal fixation is required when using these materials for interpositional grafts.

Soft tissue closure is completed in two layers. Care is taken to re-approximate the mentalis muscle. Postoperative dressings, including chin-pressure bandages, are important to minimize edema and reduce incidence of hematoma formation.

### Malar augmentation using demineralized bone

A decision is made after maxillary, mandibular, and chin repositioning as to whether cheek augmentation creating maxillary projection is aesthetically desirable. If it is, then the following technique is utilized. The senior surgeon's preference is to utilize demineralized bone when cheek augmentation and/or anterior maxillary augmentation is needed.

Two implants of demineralized bone are utilized using lyophilized, banked, perforated, demineralized bone from the Pacific Bone Bank. Over a period of time, it was determined that perforated, demineralized bone was superior to other products available on the market. An extensive experience was accumulated, starting in 1990, when a case requiring extensive scull and facial reconstruction was performed.[4] Over time, a number of different preparations of this bone coming from various donor sites was utilized according to the needs of the particular anatomical site and the adaptability of the material. It was determined that demineralized, perforated, cancellous/cortical bone harvested from the iliac crest gave the best results. Segments that are 7–8 cm × 3–4 cm × 0.6 cm in dimensions, are soaked in antibiotic solution for approximately 30 minutes until they are pliable and soft.

These pieces are then trimmed, tapered, and cut according to the desired shape for each case. This is easily done using a pair of scissors, and usually a notch is cut in the material to allow for the infraorbital nerve. The graft material is inserted through the oral incision and placed into a subperiosteal pocket created laterally over the malar-zygomatic region. The goal is to place the implant without distortion of the soft tissues and at the same time, fix it in its proper position utilizing a subperiosteal pocket laterally. It is then secured into position with 4-0 PDS or monocryl suture.

### Split calvarial bone grafting

The primary approach for cleft patients in this series when maxillary or malar augmentation was needed after definitive growth was complete, was the use of perforated demineralized bone. This gave an excellent augmentation to the deficient facial skeleton that is seen in the cleft patient, and was easy to use and gave consistent results. No other techniques were used in this series of patients. Other techniques are available and have been used by the senior surgeon in patients with other diagnoses.

The gold standard for skeletal grafting in cranial facial surgery remains cranial bone grafting.[15,16] It is not practical to obtain a cranial bone graft when treating a patient with orthognathic surgery. When performing intracranial surgery, such as correction of craniosynostosis, it is simple and easy to use cranial bone grafts, which are the senior surgeon's materials of choice. These inlay and onlay grafts do extremely well and remain the standard. Prior to the time of the use of perforated, demineralized bone grafts, the senior author utilized cranial bone grafts preferentially, at times, even in cases of Lefort I maxillary advancement. Since 1990, with the discovery of the successful use of perforated, demineralized bone by the senior surgeon, no additional cranial bone has been used for malar augmentation in the cleft patient.[2–4]

### Lamellar split bone grafting

**Figure 6:**
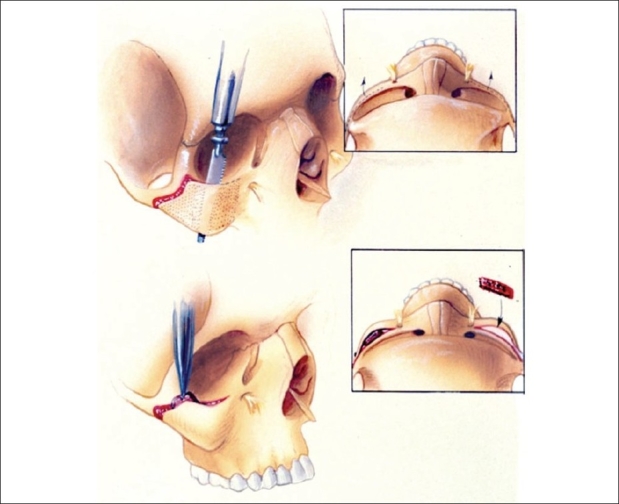
Lamellar split osteotomy technique

The lamellar split osteotomy is a surgical technique that was developed in the late 1980s and was reported in the literature in 1990.[5,6] This technique allows changing in the position of the outer bone cortex by splitting it *in situ* as you would a segment of cranial bone on the back table. The idea for this technique came to the senior surgeon when performing the splitting of segments of cranial bone. The advantage of this method is that it allows for vascularization and attachment of the muscles to the bone and still allows the reshaping of the bone to provide enhancement of the shape, angularity, and projection of the malar skeleton [Figure 6].

### Àlloplastic implants

The use of àlloplastic implants for inlay and onlay grafting of the maxilla and mandible is a technique that is used by many surgeons. These techniques have been perfected and provide a simple, “off-the-shelf” method of augmenting and contouring the shape of the face. Many materials are used and are available for this purpose. More recently, the use of prefabricated, computer-designed implants provide accurate, simple, and usually expensive solutions for the surgeon. The senior surgeon continues to look for the ideal bone replacement material that is safe and simple to use, with minimal complications.

The senior author's experience has included the use of hydroxyapatite, proplast, methyl methacrylate, polypropylene, and polyethylene materials. Any of these materials can successfully be used, some with higher infection and complication rates than others. They are not recommended in the cleft patient because of increased scarring seen in these patients with the possibility of exposure and loss.

The goal now is to use materials which become incorporated by the patient, are osteoinducting, and undergo some remodeling. For this reason and others, perforated, demineralized bone was the only material used for onlay augmentation of the maxilla and malar region in this series of patients. Osteoinduction plus remodeling provides ideal long-term results in young teenagers, and has resulted in good results with minimal complications and an infection rate of < 2%. In this series of 103 consecutive patients, none of the patients augmented in the malar region had an infection from the use of this material. There were only two minor infections in this entire series and those two were unrelated to the perforated, demineralized bone.

## RESULTS

Between 1997 and 2004, 103 consecutive patients with complete data and a diagnosis of nonsyndromic cleft lip and/or palate, and who had undergone Lefort I osteotomy, were identified by the senior author (KS).

### Preoperative

(Table 3: Preoperative Patient Demographics)

**Table 3:** Preoperative Patient Demographics

Total Patients	103
Age Range	15–41 years
	(Mean Age 18 years)
Unilateral Cleft Lip/Palate	54
Bilateral Cleft Lip/Palate	46
Isolated Cleft Palate	3
Follow-up Range	12–110 months
	(Mean Follow-up 26.2 months)
Total Number Patients in which Senior Author (KS)	
Performed Initial Cleft	
Surgery at Infancy	44
Total Number of Patients	
Planned with Same	
Orthodontist	95

Fifty-nine male and 41 female patients were seen and evaluated with an age range of 15 to 41 years (mean age: 18 years).

Fifty-four patients had a unilateral cleft lip/palate (UCLP), 46 had bilateral cleft lip/palate (BCLP) and three had isolated cleft palate. Follow-up ranged from 12 to 110 months (mean: 26.2 months). In 44 of the cases, the senior author had performed the initial cleft surgery when the patients were infants.

### Unilateral cleft lip and palate (UCLP) cases

(Table 4: Demographics Unilateral Cleft Lip and Palate Cases)

**Table 4:** Demographics: Unilateral Cleft Lip and Palate Cases

Total Cases	54
Patients with Initial Surgery Performed by KES	18
Average Number of Operations to Reach Growth	
Completion	4.5
Number of Maxillary Segments	
One-piece Maxillary Segment	41
Two-piece Maxillary Segments	10
Three-piece Maxillary Segments	3
Prior Maxillary Surgery	
Distraction Osteogenesis	1
Orthognathic Surgery	2

Of 54 cases, 20 patients had initial surgery performed by KES and each patient had an average of 4.5 operations to reach growth completion.

#### Number of maxillary segments

Forty-one cases were one-piece maxillary movements, ten cases were two-piece maxillary movements, and three were three-piece maxillary movements.

#### Previous maxillofacial surgery

One patient had previous distraction osteogenesis, and two patients had orthognathic surgery performed at another institute.

#### Planned maxillary movements

Average planned horizontal advancement was 8.6 mm (range: 4–15mm).

#### Complications

A single complication arose in this group of patients with a postoperative bleed requiring return to the operating room to obtain hemostasis. The patient did not require transfusion.

### Bilateral cleft lip and palate (BCLP) cases

(Table 5: Demographics Bilateral Cleft Lip and Palate Cases)

**Table 5:** Demographics: Bilateral Cleft Lip and Palate Cases

Total Cases	46
Patients with Initial Surgery Performed by KES	23
Average Number of Operations to Reach Growth	
Completion	7.1
Number of Maxillary Segments	
One-piece Maxillary Segment	38
Two-piece Maxillary Segments	4
Three-piece Maxillary Segments	4
Prior Maxillary Surgery	
Distraction Osteogenesis	6
Orthognathic Surgery	0

Of 46 bilateral cleft palate cases, 23 patients had initial surgery done by KES and each of this group had an average of 7.1 operations to reach growth completion.

#### Number of maxillary segments

Thirty-eight cases were one-piece maxillary advancements, four cases were two-piece maxillary advancements, and four cases were three-piece maxillary advancements.

#### Previous maxillofacial surgery

Six patients had previous distraction osteogenesis procedures performed by KES. Of these six, five were patients followed by KES since their birth. At the time of orthognathic surgery, none of these patients had any evidence of fistulae, the premaxillas were stable, and all underwent one-piece Lefort I surgery. Average horizontal advancement was 7.2 mm.

None of these patients suffered perioperative complications and none required additional operations. One patient ended up with edge-to-edge occlusion but declined further corrective surgery.

#### Planned maxillary advancement

Average planned advancement was 8.3 mm (range; 4–12 mm).

#### Complications

Two perioperative complications were noted in this group of patients. Both complications were superficial infections, one which led to failure of palatal fistula closure and relapse, requiring repeat Lefort I osteotomy.

Three patients developed gingival recession.

#### Mobile premaxilla

Seven mobile premaxillas were noted preoperatively. All surgeries in this subgroup were uncomplicated. Six of the cases resulted in a stable premaxilla postoperatively. One required a separate surgical procedure to regraft the maxilla, which resulted in complete bony union.

### Isolated cleft palate (ICP) cases

(Table 6: Demographics Isolated Cleft Palate)

**Table 6:** Demographics: Isolated Cleft Palate

Total Cases	3
Patients with Initial Surgery Performed by KES	3
Average Number of Operations to Reach Growth	
Completion	2
Number of Maxillary Segments	
One-piece Maxillary Segment	3
Two-piece Maxillary Segments	0
Three-piece Maxillary Segments	0
Prior Maxillary Surgery	
Distraction Osteogenesis	0
Orthognathic Surgery	0

Three cases were noted, all of whom had their initial surgery done by KES. Each had an average of two operations to get to growth completion. All were one-segment maxillary Lefort I advancements. None of these patients suffered complications, and none required reoperation.

None of the isolated cleft palate patients had previous maxillofacial surgery and no fistulae were present.

Average advancement in the antero-posterior plane was 5.7 mm. (range: 4–8 mm)

### Total cases

(Table 7: Total Type of Maxillary Lefort I Osteotomy Procedures)

**Table 7:** Types of Maxillary Lefort I Osteotomy Procedures

*Number of Maxillary Segments*	*UCLP*	*BCLP*	*ICP*	*Total*
One-piece Maxillary Segment	41	38	3	82
Two-piece Maxillary Segments	10	4		14
Three-piece Maxillary Segments	3	4		7
Total	54	46	3	103

Total Type of Maxillary Lefort I Osteotomy Procedures

One-piece maxillary advancement: 82 cases

Two-piece maxillary segmental advancement: 14 cases

Three-piece maxillary segmental advancement: seven cases.

Nine two-piece and two three-piece advancements were performed for fistulae that were present; modified incisions were invariably used and the iliac crest bone was grafted to the site.

In some cases, controlled osteotomies were required to alter the transverse width of the maxilla or to facilitate differential movements. In these cases, modified incisions were not used, but careful dissection of attached gingiva was performed to allow adequate exposure for interdental osteotomies. Five two-piece advancements and five three-piece advancements were performed in this way.

No teeth were damaged in any procedure and 99% of the procedures resulted in a stable arch form. As mentioned previously, one case required regrafting to stabilize a postoperatively unstable premaxilla.

### Adjunctive procedures

Fifty-four patients had onlay demineralized bone placed to augment the zygomatic prominences. Fifty-one had bilateral sagittal split osteotomies of the mandible, 32 had genioplasty procedures, 24 had inferior turbinate resections, seven had buccal fat excision, and one had liposuction of the neck.

### Relapse rate

Thirteen patients displayed significant relapse. All relapses were clinically evident within a year of follow-up. Due to significant relapse, nine patients underwent “redo” orthognathic surgery to successfully correct the deformity.

Results were statistically analyzed and there were no significant variables although the presence of preoperative fistulae approached significance (*P* = 0.11).

#### UCLP

Five patients (10% of UCLP) developed relapse with recurrence of negative overjet/overbite. Of these five patients, three had repeat orthognathic procedures. The remaining two patients accepted the less-than-ideal edge-to-edge occlusion.

#### BCLP

Eight patients (17% of BCLP) displayed evidence of relapse, of which six required repeat Lefort I procedure and two accepted the relapsed occlusion.

### Reoperative rate

A 11% re-operative rate was noted with two patients requiring further operations to close fistulae. One patient required an operation to close a fistula, graft a mobile premaxilla, and correct relapse. Eight patients required an additional procedure for relapse alone. Seven of the reoperated patients had BCLP.

### The long-term outcomes

Four hundred fifty consecutive cleft patients with complete data and who had been treated from 1969 to 2007, were reviewed. In unilateral cleft patients, 30% required orthognathic surgery. In bilateral cleft patients, 40% underwent orthognathic surgery to achieve facial balance and harmony after completion of growth.

**Figure 7:**
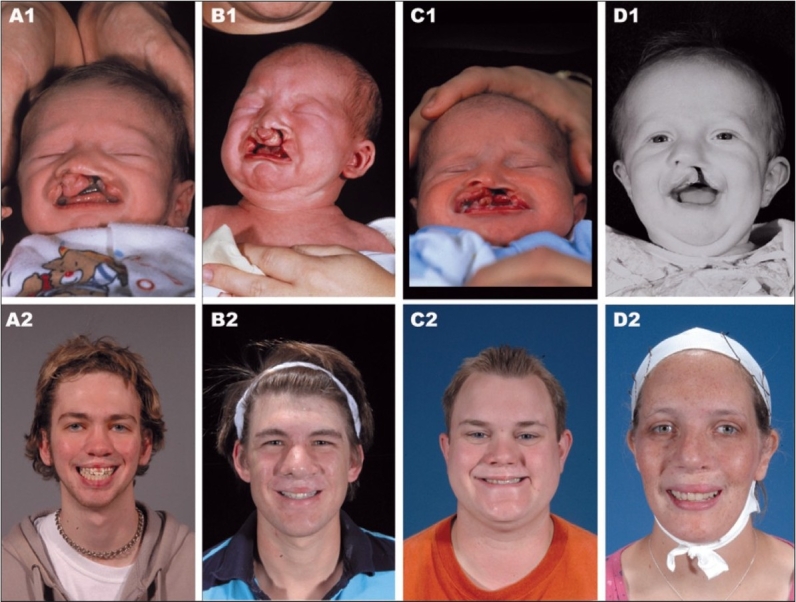
Four cases treated from infancy through completion of growth. Case 1. (A1) Bilateral complete cleft lip/nose, alveolus and palate at three months of age. (A2) 18 years of age. Case 2. (B1) Bilateral complete cleft lip/nose, alveolus and palate at three months of age. (B2) 20 years of age. Case 3. (C1) Unilateral complete cleft lip/nose, alveolus and palate at three months of age. (C2) 23 years of age. Case 4. (D1) Unilateral complete cleft lip/nose, alveolus, and palate at three months of age. (D2) 19 years of age

The outcomes of the representative cases are included here [Figures 7–11]. These figures illustrate one unilateral cleft and three bilateral cleft cases that were treated from infancy through the completion of growth.

**Figure 8A:**
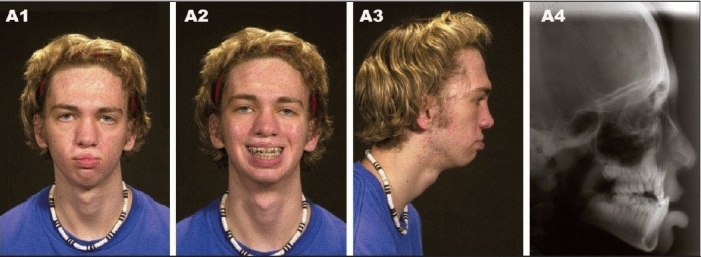
(A) A 17 year-old patient with bilateral complete cleft lip/nose, alveolus and palate after completion of growth and before orthognathic surgery. (A1) Frontal view (A2) Frontal smiling (A3) Right lateral view (A4) Preoperative lateral cephalometric roentgenogram

**Figure 8B:**
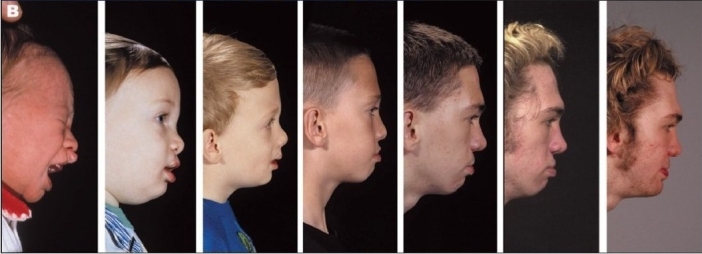
(B) Right lateral view from infancy until completion of treatment

**Figure 8C:**
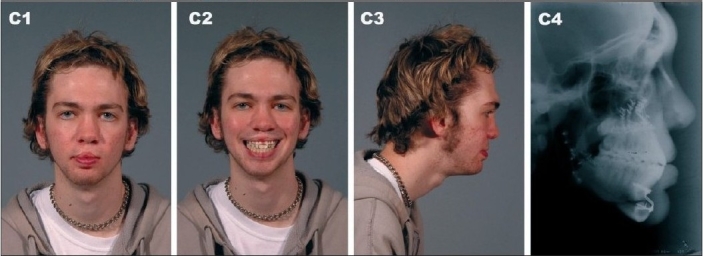
(C) 18 years of age showing long term outcome after 3-piece Lefort I osteotomy with maxillary advancement, bilateral mandibular ascending ramus sagittal split osteotomy and genioplasty. (C1) Frontal view (C2) Frontal smiling (C3) Right lateral view (C4) Postoperative lateral cephalometric roentgenogram

**Figure 9A:**
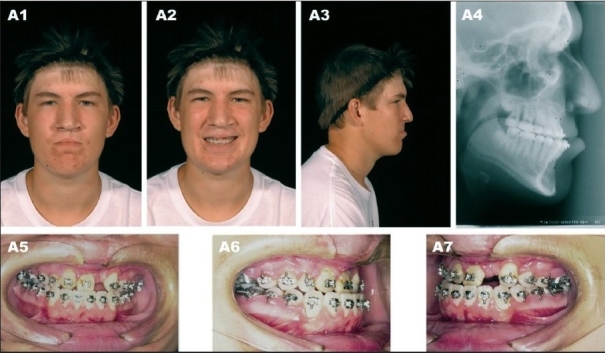
(A) A 17 year-old patient with bilateral complete cleft lip/nose, alveolus and palate after completion of growth and before orthognathic surgery. (A1) Frontal view (A2) Frontal smiling (A3) Right lateral view (A4) Preoperative lateral cephalometric roentgenogram (A5) Frontal view with orthodontic brackets (A6) Right oblique view with orthodontic brackets (A7) Left oblique view with orthodontic brackets

**Figure 9B:**
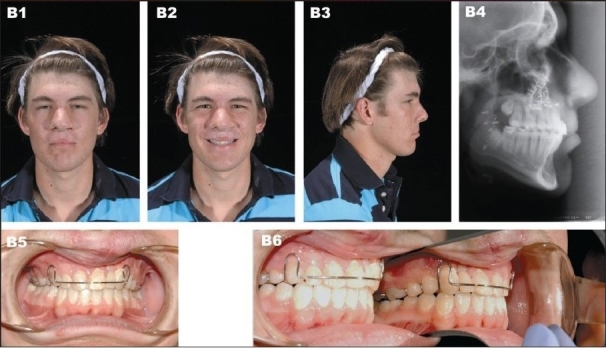
(B) Patient at 20 years of age demonstrating long-term outcome after a four-piece Lefort I osteotomy with maxillary advancement plus bilateral mandibular ascending ramus sagittal split osteotomy. (B1) Frontal view (B2) Frontal smiling (B3) Right lateral view (B4) Postperative lateral cephalometric roentgenogram (B5, B6) Occlusion after completion of treatment

**Figure 10A:**
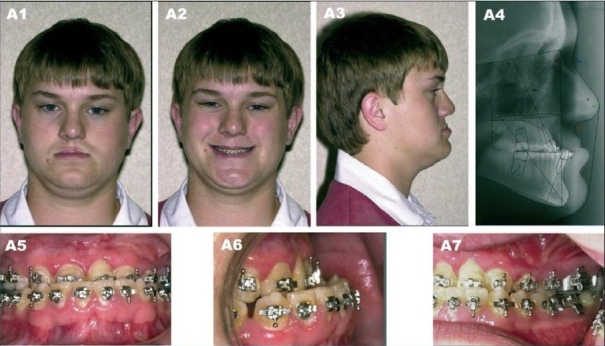
(A) A 16 year-old patient with unilateral complete cleft lip/nose, alveolus and palate after completion of growth and before orthognathic surgery. (A1) Frontal view (A2) Frontal smiling (A3) Right lateral view (A4) Preoperative lateral cephalometric roentgenogram (A5) Frontal view with orthodontic brackets (A6) Right oblique view with orthodontic brackets (A7) Left oblique view with orthodontic brackets

**Figure 10B:**
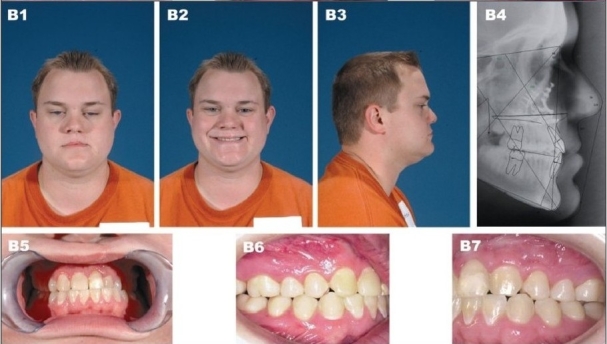
(B) Patient at 23 years of age demonstrating long-term outcome after Lefort I osteotomy with maxillary advancement. (B1) Frontal view (B2) Frontal smiling (B3) Right lateral view (B4) Postperative lateral cephalometric roentgenogram (B5, B6, B7) Occlusion after completion of treatment

**Figure 11A:**
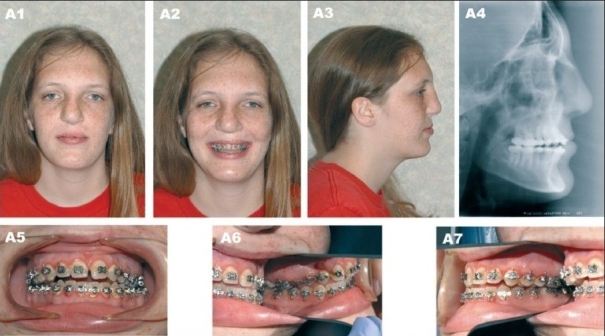
(A) A 16 year-old patient with unilateral complete cleft lip/nose, alveolus and palate after completion of growth and before orthognathic surgery. (A1) Frontal view (A2) Frontal smiling (A3) Right lateral view (A4) Preoperative lateral cephalometric roentgenogram (A5) Frontal view with orthodontic brackets (A6) Right oblique view with orthodontic brackets (A7) Left oblique view with orthodontic brackets

**Figure 11B:**
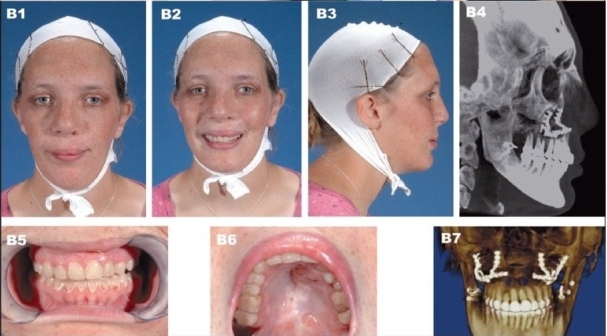
(B) Patient at 19 years of age demonstrating long-term outcome after two-piece Lefort I osteotomy with maxillary advancement, bilateral mandibular ascending ramus sagittal split osteotomy, and onlay demineralized bone grafting to bilateral cheeks. (B1) Frontal view (B2) Frontal smiling (B3) Right lateral view (B4) Postperative lateral cephalometric roentgenogram (B5, B6) Occlusion after completion of treatment (B7) 3D CT image showing the final occlusion and internal semi-rigid fixation

## DISCUSSION

The surgical approach to the cleft deformity requires an ongoing treatment plan from infancy to adulthood.[17] This paper deals with one surgeon's experience trying to make improvements in the treatment of the cleft deformity. Of course, each patient is different and surgical planning and techniques must be individualized in order to achieve optimal facial balance and harmony. The protocols are a rough guide to the individualized treatment necessary in each case to optimize the result. The goal is to achieve balance and harmony and attractiveness of the face, providing aesthetic form and normal function. In order to achieve this goal, the surgeon must aim for the construction of a projecting skeletal base, which then overcomes some of the classic stigmata of the cleft deformity.

The achievement of a balanced skeletal base necessitates more than correcting the occlusion. The patient is assessed and treated during the growth of the face by the surgeon and orthodontist until growth is complete. During growth, the teeth are leveled and aligned throughout the mixed dentition and permanent dentition stages. The growth of the jaws is allowed to occur, and individual leveling and aligning is performed without an attempt to compensate for the deficiency in maxillary growth. The treatment plan is determined in each case when growth is complete. It is then most obvious when orthodontics alone will not give an optimal facial projection and aesthetic balance.

Dental occlusion alone should not dictate the treatment plan. In our protocol, the face and aesthetics were the primary concerns, and the patient's occlusion was then adjusted accordingly. The treatment planning revolved around placing the maxilla in the optimal horizontal and vertical positions to achieve attractiveness when the patient smiled. The goal was for a patient to show a full set of upper teeth when smiling, with protrusion of the upper vermilion of the lip out over the lower lip at rest. If the skeletal base of the maxilla and mandible was deficient or out of optimal position, it was corrected after growth was completed. This frequently was still not enough to achieve optimal facial balance and harmony and adjunctive measures were undertaken.

A projecting convex face corrects the cleft deformity and provides a foundation for correction of the abnormal skeleton. At the same time, the goal of an attractive face is attained by allowing redraping of the soft tissues of the face and the creation of a new, balanced, skeletal facial harmony. The methods and techniques used today were not available 40 years ago, and early results were not nearly as good as those that can be achieved today. In the future with further developments, the results will be even further improved. All good results in cleft surgery require the surgeon to work carefully with an interdisciplinary team. A close working relationship with the orthodontist is integral to achieve optimal results.

In the cleft patient, it was found that the optimal malar projection could most conveniently and safely be obtained with minimal complications using perforated, demineralized bone. In all 103 patients treated in this series, those that needed augmentation were all treated in the same fashion. Demineralized bone is ideal because it is osteoinductive with minimal resorption, but allows for some remodeling and smoothing over time, providing long-term consistent results. Other techniques have been described in earlier studies and can still be used according to the diagnoses and treatment plan. In maxillary and malar augmentation, demineralized bone is much easier to use and provides a more consistent result than does cranial bone. Although, cranial bone remains the gold standard facial bone grafting material, demineralized bone has been proven by the senior surgeon to be a superior material for augmentation of the maxilla and zygoma in the cleft patient.

The split lamellar technique was developed by the senior surgeon to be used in cases where the occlusion was normal but the mid-face and malar regions were deficient. One of the first cases was performed in a cleft patient who had received orthodontic treatment elsewhere, but had been an original cleft patient of the senior surgeon.

The orthodontist had ignored facial balance principles, and the teeth were orthodontically moved to occlusion with disregard to the maxillary deficiency, resulting in a normal occlusion with a dish face deformity. Orthodontics performed without regard to facial aesthetic principles in the cleft patient will result in the dish-face, or at least, a straight profile, contributing to an abnormal facial balance. In this particular case, the patient did not want to undergo another year or two of orthodontics, which would have been required in order to achieve proper preparation for maxillary and/or mandibular surgery.

**Figure 12:**
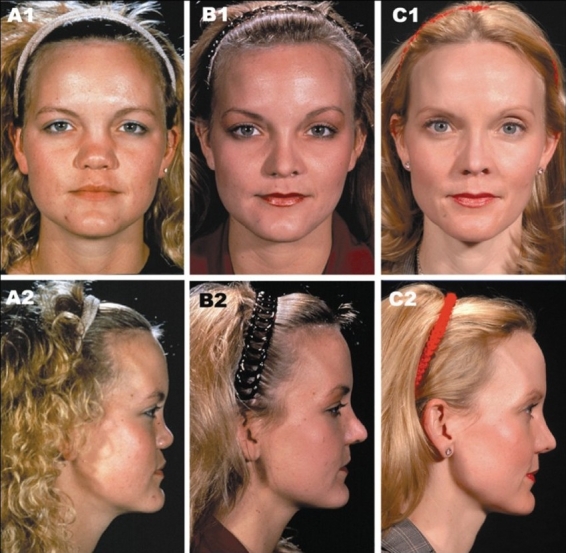
Long-term outcome of malar augmentation using the lamellar split osteotomy in a non-cleft patient. (A1, A2) Frontal and lateral view of a noncleft patient with flat malar region before lamellar split osteotomy. (B1, B2) one year, after lamellar split osteotomy. (C1, C2) 13-year, long-term outcome after lamellar split osteotomy

The lamellar split technique allows splitting of the outer table of the malar bone, extending to the inferior orbital nerve and leaving muscular and periosteal attachments intact. These attachements protect the anterior from resorption. The anterior table is moved and reshaped to provide proper facial projection. This technique requires expert execution but provides a good alternative method for achieving facial projection in the patient with maxillary deficiency in normal occlusion [Figure 12]. This was the 13-year long-term outcome of malar augmentation using the lamellar split osteotomy in a noncleft patient. She was provided additional cranial bone at the time, including nasal reconstruction with cranial bone to the dorsum and septal cartilage for tip projection with excellent long-term results.

### Genioplasty

At the time of maxillary and mandibular surgery, a sliding genioplasty can often significantly improve the facial aesthetic balance by adjusting the lower facial height and projection. The planning for a genioplasty is suggested preoperatively by facial aesthetics, and computer-based confirmation can be obtained with facial profile projections. The ultimate decision is made on the operative table by the surgeon after the jaws have been moved and fixed into the final position with internal rigid fixation. In order to achieve facial projection, a prominent chin is desirable in both the male and female patients. In the female patient, a subtler projection and narrower chin, as seen in the ancient Egyptian Queen Nefertiti, is optimal. The Egyptians, Greeks, and Romans achieved and demonstrated facial balance in most of their facial sculptures, and had an appreciation for facial balance and harmony. This same concept holds true today for our cleft patients. Achieving a functional occlusion alone without regard to facial aesthetics, is an antiquated goal of orthognathic surgery. We hope that more of our colleagues will strive for excellence in the comprehensive care of cleft patients, providing a much more acceptable face to society and psychosocial rehabilitation of the patient. The habilitation of the cleft patient remains a challenge for the interdisciplinary team. It is one of the main reasons that a cleft surgeon continues his/her quest for perfection. The techniques presented in this paper were performed on 103 cases. The methods and philosophy used are the examples of one surgeon who is driven by perfection and the desire in each case, to achieve as optimal a result as possible.

### Internal semi-rigid fixation

After Lefort I maxillary osteotomy, four-point wire fixation is used before the application of internal semi-rigid fixation. We preferred to use the KES-designed 0.6 thickness titanium “T” plates, instead of using heavier plates. Two of these plates are secured on each side of the maxilla and IMF elastics are used postoperatively. This method may have contributed to the relapse rate of 10% in the unilateral cleft lip patients and 17% in the bilateral cleft patients. However, it is felt that relapse is frequently limited to a few millimeters in most patients. Final adjustments are easier to make with this approach. For one to accurately assess this hypothesis, it would be necessary to study another series with heavier internal rigid fixation.

### Complications

The performance of the surgery was by an experienced, highly skilled operative team. Surgical planning and ongoing orthodontic treatment was by a senior orthodontist (EG). The complications other than relapse were minimal, with one postoperative bleeding episode that required return to the operating room, but not transfusion. Two minor infections were noted, which were treated with antibiotics and required no surgical intervention. Even though nearly half of these patients had ideal treatment from the time of birth, the total group had an overall relapse rate of 10% in the UCLP and 17% in the BCLP group. Three patients in the UCLP group and six patients in the BLCP group underwent secondary surgery. The relapse rate for the senior surgeon's series of cleft patients is higher than the relapse rate in noncleft patients and nonsyndrome patients operated on with the same procedures.

Cleft patients continue to present an ongoing treatment challenge, even with the known and proven procedures available today. Cleft surgery requires an expert team performing ongoing treatment in order to achieve optimal outcomes. The concept of treating cleft patients in the developing world with mission surgery without proper follow-up and team care results in suboptimal results. All efforts for the treatment of global cleft patients should be directed towards the development of sustainable teams. These teams can treat large volumes of patients and develop extensive expertise in the necessary ongoing optimal interdisciplinary approach to the cleft patient.
